# Elevated Resting State Gamma Oscillatory Activities in Electroencephalogram of Patients With Post-herpetic Neuralgia

**DOI:** 10.3389/fnins.2018.00750

**Published:** 2018-10-23

**Authors:** Rui Zhou, Jing Wang, Wenjing Qi, Feng-Yu Liu, Ming Yi, Huailian Guo, You Wan

**Affiliations:** ^1^Neuroscience Research Institute, Peking University, Beijing, China; ^2^Department of Neurobiology, School of Basic Medical Sciences, Beijing Institute For Brain Disorders, Capital Medical University, Beijing, China; ^3^Department of Rehabilitation Medicine, Peking University Third Hospital, Beijing, China; ^4^Department of Neurology, Peking University People’s Hospital, Beijing, China; ^5^Key Laboratory for Neuroscience, Ministry of Education/National Health Commission, Peking University, Beijing, China

**Keywords:** chronic pain, post-herpetic neuralgia, electroencephalogram, gamma rhythm, power spectrum

## Abstract

In acute and ongoing pain, the spontaneous oscillatory activity of electroencephalogram (EEG) has been characterized by suppression of alpha band oscillations and enhancement of gamma band oscillations. In pathological chronic pain which is more severe and common in clinic practice, it is of great interest to investigate the oscillatory activity especially at the broad gamma frequency bands. Our present study explored the resting state oscillatory activities of EEG in patients with post-herpetic neuralgia (PHN) over 3 months which is a typical neuropathic pain model in clinical researches. It was found that the PHN patients showed anxiety and depression revealed by Beck Anxiety Inventory (BAI) and Beck Depression Inventory (BDI) examinations. Power spectrum analysis revealed that the power at gamma frequency band (from 40 to 70 Hz) of EEG was significantly higher in the PHN patients, and positively correlated with pain intensity, anxiety, and depression indexes. Further, increased gamma activity derived from the prefrontal cortex and the cerebellum were revealed by cluster-based sensor level and the beamforming source level analyses. These results suggest the enhanced gamma oscillatory activity in the prefrontal cortex and cerebellum is a characteristic marker in chronic neuropathic pain patients.

## Introduction

Brain oscillation is rhythmic electrical activity and has distinct functions at different frequencies in various neural processing. It has been shown that oscillatory activities at alpha, beta, and gamma frequency bands are involved in short-lasting acute pain. Decreased alpha power together with increased beta and gamma power of electroencephalogram (EEG) was detected after painful stimulation (Mouraux et al., 2003; Ploner et al., 2006; May et al., 2012; Hu et al., 2013). The power at gamma frequency band (40–70 Hz) increased in experimental acute pain processing in healthy subjects and animals (Gross et al., 2007; Tiemann et al., 2010; Schulz et al., 2011; Wang et al., 2011; Zhang et al., 2012; Liu et al., 2015). For tonic pain in human beings, gamma power was also increased (Peng et al., 2014; Schulz et al., 2015; Li et al., 2016), and notably, the increased gamma power was positively correlated with subjective pain intensity.

Chronic pain, especially neuropathic pain, is more severe and common in clinic practice. Pathological pain severely impairs the life quality of patients. It is characterized as persistent and long-lasting pain (Merskey and Bogduk, 2012), which is distinct from the short-lasting experimental acute pain and tonic pain. Unlike the experimental and tonic pain, information regarding the brain oscillatory activity in chronic pain is few.

Thalamocortical dysrhythmia (TCD) refers to a persistent oscillatory imbalance which manifests increased spectrum power at theta and gamma band (Llinas et al., 1999). TCD is considered as the underlying mechanism of chronic pain. Previous researches about oscillatory activities ranged from 2 to 45 Hz found that patients with neuropathic pain exhibited increased power at theta frequency band (Sarnthein et al., 2006; Boord et al., 2008; Jensen et al., 2013; González-Roldán et al., 2016; Fallon et al., 2018). Recently, power at narrow low gamma band (30–48 Hz) was found to be increased in rats with inflammatory chronic pain (Wang et al., 2016) and in fibromyalgia patients (Lim et al., 2016). However, the activity of broad gamma frequency band, from 40 to 70 Hz, was known very limited. Thus, it is of interest to investigate oscillatory activities over all frequency bands (from 2 to 70 Hz) in chronic pain patients.

Post-herpetic neuralgia (PHN) is a common type of neuropathic pain, which lasts beyond the healing of rash caused by herpes zoster (Bowsher, 1999). It is characterized by continuous burning or aching pain, or intermittent sharp pain and considered as a prototypical human chronic neuropathic condition, because such patients exhibit multiple peripheral and central manifestations of neuropathy. The etiology of PHN made it an appropriate model for neuropathic pain research.

Therefore, in our present study, we investigated the oscillatory activities of EEG, their spatial distribution and source location, and its correlation with pain symptoms in chronic PHN patients.

## Materials and Methods

### Subjects

Fourteen patients (age 64.4 ± 6.9, range 55–74 years old, 10 women, 4 men) with PHN were recruited at the outpatient clinic of Peking University People’s Hospital. PHN was diagnosed based on medical history and physical examination according to diagnostic criteria (Gupta and Farquhar Smith, 2012). The inclusion criteria were that all patients were over 18 and below 75 years old with constant pain for at least 3 months. Exclusion criteria included no migraine, tension headache, peripheral neuropathy, osteoarthritis, no other causes of acute and chronic pain, and no other chronic diseases and neurological diseases which might affect the EEG. For the consideration of treatment, the PHN patients were not free of medication. Medication with pregabalin, carbamazepin, or diazepam was included, but the opioid analgesic was excluded in this study. Two patients used tramadol but stopped more than 3 days before the EEG recordings (following doctor’s advice but not for the sake of our research purpose). The demographics, medical history, and clinical characteristics of patients were summarized in **Table 1**.

**Table 1:** Demographic and clinical description of each PHN patient.

No.	Pain location	Pain side	Drug history	Pain duration	VAS	BAI	BDI
1	Leg	Left	None	1 year	5	5	17
2	Chest and back	Right	None	5 months	2	3	2
3	Forehead and vertex	Right	Gabapentin and tramadol	4 months	3	9	5
4	Face and tongue	Left	Citalopram and zopiclone	2 years	2	4	4
5	Back and abdomen	Left	None	3 months	3	7	1
6	Abdomen	Right	Zolpidem	6 years	5	15	12
7	Shoulder and arm	Left	None	3 months	7	12	9
8	Chest	Left	None	4 months	9	9	5
9	Chest, back, and arm	Left	Carbamazepine	10 months	9	13	14
10	Low back, vulva, and leg	Right	Gabapentin	3 months	4	3	0
11	Chest and back	Right	Pregabalin and diazepam	9 months	8	29	15
12	Low back and buttocks	Left	Gabapentin and tramadol	9 months	4	9	6
13	Low jaw	Left	Selegiline	5 months	3	17	4
14	Arm	Right	None	3 months	6	25	14

*F, female; M, male; VAS, visual analog scale; BAI, Beck anxiety inventor; BDI, Beck depression inventory.*

The healthy controls (HC) group comprised 14 healthy participants matched by age (65.2 ± 7.9) and gender. Both patients and healthy participants gave their written informed consent to use the data for scientific purposes. All subjects were right handed and the education level was above junior high school (3 bachelors, 13 senior high schools, and 8 junior high schools). Procedures were approved by the Ethics Committee of Peking University People’s Hospital.

### Clinical Evaluation of Pain, Depression, and Anxiety in PHN Patients and Control Subjects

All subjects filled the Short-Form of McGill Pain Questionnaire (SF-MPQ) (Melzack, 1987). The SF-MPQ included a verbal Visual Analog Scale (VAS) (0 = no pain, 10 = maximum imaginable pain). Depression scores and anxiety scores were assessed using the Beck Depression Inventory (BDI) (Beck et al., 1996) and the Beck Anxiety Inventory (BAI) (Beck et al., 1988). The BAI contained 21 questions, each answer was scored on a scale value of 0 (not at all) to 3 (severely). Higher total scores indicated more severe anxiety symptoms. BDI consisted of 21 questions about how the subject had been feeling in the last week. Each question had a set of at least four possible responses, ranging in intensity. When the test was scored, a value of 0 to 3 was assigned for each answer and then the total score was compared to a key to determine the depression’s severity. Higher total scores indicated more severe depressive symptoms. All questionnaires were asked to be answered 1 h prior to the EEG recording.

### EEG Recordings

Net station 5.3 software (Electrical Geodesics Inc., Eugene, United States) was used for the EEG recording. The subjects sit relaxed in a dim room to prevent from disturbing and weared the 128-channel electrode cap suitable for his/her head size. Electrode CPz served as the common reference. All electrodes impedance were below 50 kΩ. The sampling rate was 500 Hz. Twenty minutes of resting EEG were recorded during eyes closed. During the recording, the subject remained awake to avoid sleep.

### EEG Data Pre-processing

The raw EEG data were preprocessed offline using MATLAB-based EEGLAB toolbox (The Mathworks^1^). Large muscle or eye movement artifacts were removed using independent component analysis (ICA), which showed a large electrooculogram (EOG) channel contribution and a frontal channel distribution (Delorme and Makeig, 2004). Bad channels with huge jumps or completely flat were manually removed and then replaced by spline interpolation implemented in EEGLAB. Time frames with the amplitude exceeding 100 μV were rejected. The scalp EEGs were then re-referenced to the mean of the signals recorded at the average reference. The band-pass filter was set between 1 and 70 Hz and notch filter was applied to remove the 50 Hz line noise artifacts. The procedure resulted in at least 400 s EEG data of each subject for further analysis (data length = 723.8 ± 76.7 s). No significant difference of length between two groups [*t*_(26)_ = 1.26, *p* > 0.05].

### Power Spectrum

Data were processed with custom-made scripts using MATLAB version R2012a (The Mathworks, Natick, MA, United States) and FieldTrip. Power between 1 and 70 Hz in step of 1 Hz was estimated using the multitaper method (Mitra and Pesaran, 1999). Data were segmented into epochs of 2 s with a sliding window of 1 s. Slepian tapers were used to obtain a frequency smoothing of 3 Hz, and the power was averaged over segments and tapers.

### Channel Level Analysis

Topographical distribution of gamma power was plotted over channels. The non-parametric statistical significance of the difference between the two conditions was evaluated using a cluster-based randomization test (Maris and Oostenveld, 2007). Clusters showing differences between Control and Patient groups were formed along the channel and tested for significance. Briefly, a *t*-statistic at each channel was calculated. The neighbor channels with *t*-value bigger than the 95th percentile were selected and calculated as the maximum sum of each cluster. Then 500 times permutation test was conducted between two groups. The Monte-Carlo significance probability was the proportion of random partitions resulted in higher cluster-statistics than that of the actual data.

### Source Analysis

To localize the sources of gamma oscillatory activities, we used the dynamic imaging of coherent source (DICS) beamforming method (Gross et al., 2001) implemented in the MATLAB toolbox FieldTrip (Oostenveld et al., 2011). The leadfield matrix was computed for an 8-mm 3D grid using the boundary element method volume conduction model, derived from the MNI template brain provided by FieldTrip. Cross-spectral density matrices were computed for gamma frequency. By using DICS, a spatial filter was created based on cross-spectral density matrices of both Control and Patient group. For statistical analysis of neuronal activity, a *t*-test was performed on the result of each grid point to estimate the signal change between groups across subjects. Subsequently, *t*-values were transformed to *Z*-scores, MNI-coordinates of peak voxel were transformed into Talairach-coordinates (Talairach and Tournoux, 1988) and were fed into the Talairach Daemon 4 for classification purpose.

### Phase–Amplitude Coupling

Phase–amplitude coupling (PAC) was estimated by the method proposed by Canolty et al. (2006) to detect the phase–amplitude coupling and to assess the intensity of the coupling (Tort et al., 2010). The procedure was as in our previous report (Wang et al., 2016). EEG signals were firstly filtered at frequencies from 2 to 20 Hz and from 40 to 70 Hz with step of 1 Hz by FIR filter. Then, Hilbert transformation was applied to extract their amplitude and phase. We constructed a complex-valued signal [ϕ_L_(n), a_H_(n)] with low frequency phase time series ϕ_L_(n) and high frequency amplitude time series a_H_(n). The modulation index was obtained by measuring the degree of asymmetry of the probability density function of the complex-valued signal. The modulation index of the signal was compared with that of 100 surrogate data generated by shifting the amplitude time series to obtain the significance of coupling. The significant PACs with *p* < 0.05 were kept while those with *p* > 0.05 were set to zero.

### Statistics

Student *t*-test was used to compare the difference between the two groups. The Cohen’s *d* was calculated for estimating the effect size. Spearman correlation was applied to calculate the pain rating correlation with psychological behavior scores. Pearson correlation and linear regression were applied to calculate the gamma power correlation with pain intensity and related symptoms. BAI scores were controlled by logistic regression. The significance level was set at α = 0.05.

## Results

The pain rating and psychological behavior scores have been listed in **Table 2**. As revealed by independent *t*-test, PHN patients had significantly higher VAS pain ratings and BAI scores compared with healthy Controls (for VAS pain rating *p* < 0.001, for BAI score *p* < 0.001) indicating the chronic neuropathic pain and anxiety. Spearman correlation analysis further showed that the BAI score was positively correlated with VAS rating in PHN patients (*r* = 0.56, *p* < 0.05) but not in healthy Controls.

**Table 2:** Pain symptoms, depression, and anxiety scores in PHN patients.

	Patient (*n* = 14)	Control (*n* = 14)
VAS	5.0 ± 2.4^∗∗∗^	0
BAI	11.4 ± 8.0^∗∗∗^	2.3 ± 2.2
BDI	7.6 ± 5.7	4.9 ± 3.4
VAS & BAI	*r* = 0.56^∗^	*r* = 0
VAS & BDI	*r* = 0.67^∗^	*r* = 0

*^∗∗∗^p < 0.001; ^∗^p < 0.05. BAI & VAS: correlation between VAS and BAI. BDI & VAS: correlation between VAS and BDI.*

### Increase of Gamma Power in PHN Patients

**FIGURE 1:**
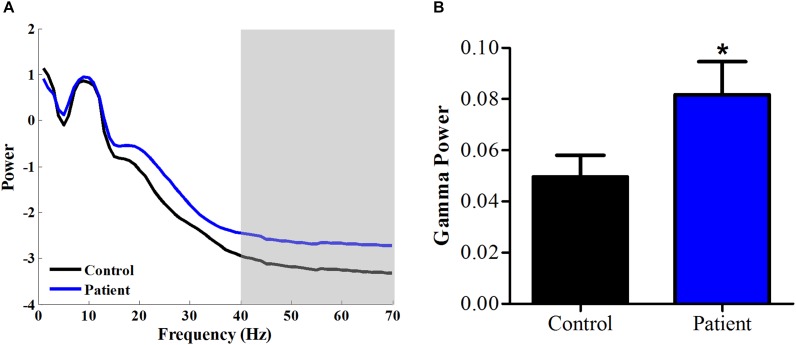
Power spectrum at high frequency increased in PHN patients. **(A)** Averaged power spectrum across all channels from 1 to 70 Hz in PHN patients and healthy controls. **(B)** Gamma power (40–70 Hz marked as gray area in panel **A**) was significantly higher in PHN patients. Power scale is μV^2^. ^∗^*p* < 0.05.

We calculated the power spectrum of spontaneous EEG from 1 to 70 Hz for both healthy Control and PHN patients. As shown in **Figure 1A**, the power at 1–40 Hz averaged across all channels was similar between Patient group and Control group, while the power at 40–70 Hz was significantly higher in Patient group compared with that in healthy Control group [*t*_(26)_ = 2.08, *p* < 0.05, Cohen’s *d* = 0.79] (**Figure 1B**), indicating that the gamma power was enhanced in PHN patients.

Since the BAI scores were different in the Patient group from the Control group, we then compared gamma power after controlling the BAI scores. It showed that the gamma power was not significantly different any more (*p* = 0.14), suggesting that the gamma power was more related to anxiety symptoms.

**FIGURE 2:**
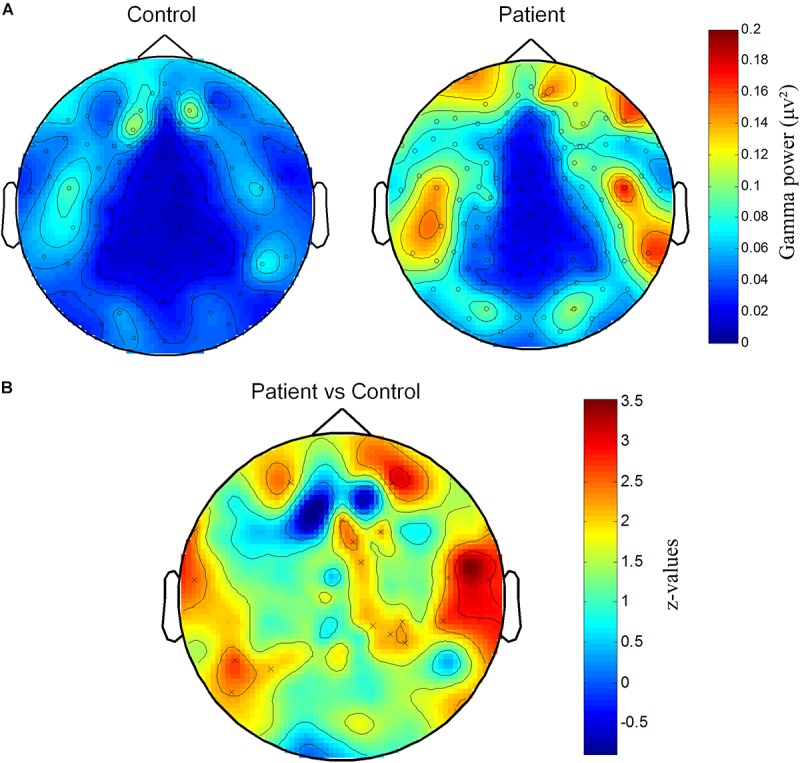
Spatial distribution of increased gamma power at channels on parietal and temporal areas. **(A)** Topography of gamma power for Patient and Control groups. Gamma power (μV^2^) is color-coded. **(B)** Significantly different distribution of gamma power between Patient group and Control group. *Z*-values representing the statistical comparison between two groups are color-coded. “×” marks the cluster that significantly higher in Patient group.

We further explored the difference of gamma activity in spatial distribution and plotted the topographic distribution of gamma power in both groups. As shown in **Figure 2A**, the gamma power (40–70 Hz) was higher in several channel locations. In order to find the areas with different gamma between groups, we compared gamma power of each channel between two groups with the *t*-test. Compared with permutation, a group of neighbor channels with sum bigger 95th *t*-value was then considered significant. Statistical analysis showed that several channels at right parietal and temporal regions, left temporal and prefrontal regions had higher gamma activities in Patient group than in Control group (**Figure 2B**).

**FIGURE 3:**
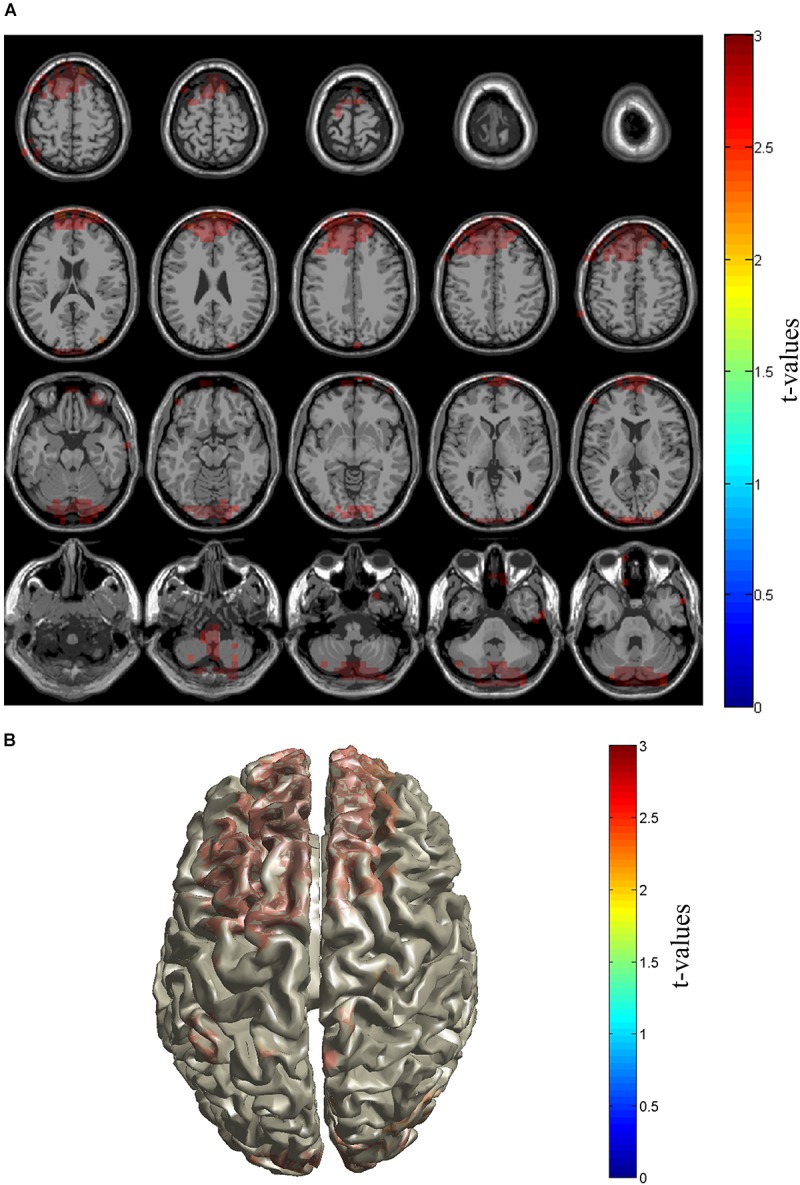
Source distribution of gamma power. Sources with enhanced gamma power in PHN patients were shown in slice **(A)** and brain cortex **(B)**. Sources locate in the prefrontal cortex including dorsolateral and medial prefrontal cortices as well as cerebellum area. The *t*-values are color-coded and only significant changes are displayed.

Then, we investigated the source location of the increased gamma activities in patients at the source level. By Beamforming source level analysis, it was found that the gamma powers in bilateral dorsolateral cortex, medial prefrontal cortex, median and anterior cingulate gyri, and the cerebellum were significantly higher in Patient group than in Control group (**Figures 3A,B**).

### Gamma Power Correlation With Pain Intensity, Depression, and Anxiety

**FIGURE 4:**
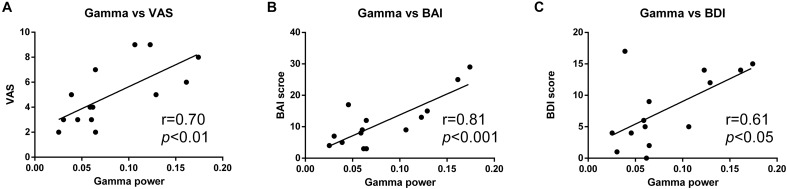
Positive correlation between gamma power and pain ratings. Gamma power was positively correlated with VAS rating **(A)**, BAI score **(B)**, and BDI score **(C)**.

We further analyzed the relationship between the increased gamma activity and the pain intensity. It was shown that gamma power was positively correlated with VAS rating in PHN patients (γ = 0.70, *p* < 0.01). Linear regression showed that gamma power could predict 48% changes of VAS (*R*^2^ = 0.48, *p* < 0.01) (**Figure 4A**). This strong correlation indicates the more increased gamma power, and the more severe pain.

Moreover, the gamma power was also strongly correlated with BDI (γ = 0.61, *p* < 0.05) and BAI (γ = 0.81, *p* < 0.001) scores, and could explain 37 and 64% variability of BDI and BAI, respectively (*R*^2^ = 0.37; *R*^2^ = 0.64) (**Figures 4B,C**).

### Gamma Power Reduction After Pain Relief: A Case Report in One PHN Patient

**FIGURE 5:**
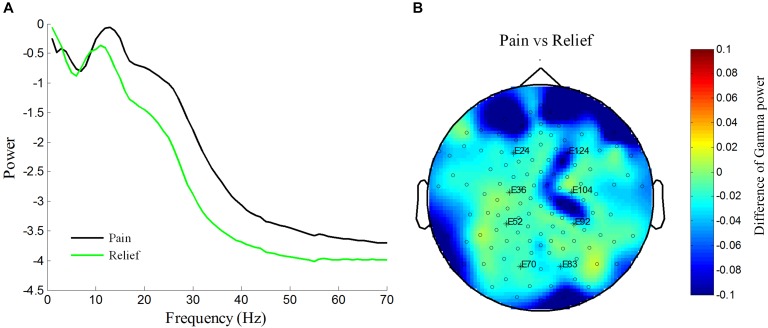
Reduction of increased power spectrum in one patient after pain relief in patient No. 10. Averaged power spectrum across all channels from 1 to 70 Hz before and after pain relief **(A)**. Power scale is μV^2^. The difference of topography of gamma power under pain condition vs. pain relief condition **(B)**. Difference of gamma power between pain and relief condition are color-coded.

One of the PHN patients (No. 10 patient in **Table 1**) was free of pain after the treatment. We took the advantage of self-comparison and measured the EEG of this patient again. By analyzing the power spectrum and power topographic distribution, we found that the power of oscillation (15–70 Hz) was reduced in the pain-free state compared with the pain state. Interestingly, the reduced gamma power was over prefrontal and parietal areas where was the similar location of the enhanced gamma power in PHN patients (**Figures 2B**, **5**). This result, although from only one case, suggests a reduction of gamma power after pain relief.

### Phase-Amplitude Coupling Between Low Frequency and Gamma Frequency Activities

**FIGURE 6:**
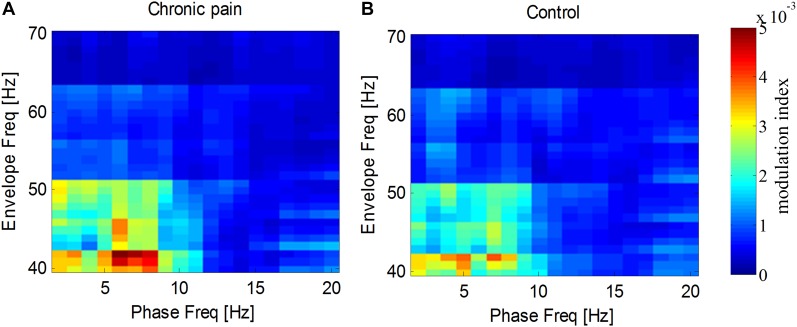
Modulation of gamma amplitude by phase at low frequencies. Gamma amplitude was modulated by phase at 2–8 Hz in PHN patients **(A)** and in healthy controls **(B)**. Modulation index are color-coded.

As the amplitude of gamma frequency is usually modulated by the phase of low frequency, we measured the phase–amplitude coupling between gamma frequency and other frequencies. It was found that the spontaneous gamma amplitude, especially from 40 to 55 Hz, was modulated by the phase of delta and theta frequency (1–8 Hz) for both groups (**Figure 6**). While, neither the theta–gamma coupling nor delta–gamma coupling in PHN group was significantly different from that in the Control group [for theta–gamma coupling, *t*_(26)_ = 0.66, *p* = 0.52, for delta–gamma coupling *t*_(26)_ = 0.19, *p* = 0.85].

## Discussion

We found that the gamma band power was increased in chronic pain patients with PHN and the enhanced gamma activity located mainly over the prefrontal cortex and cerebellar areas. Interestingly, the enhanced gamma power was positively correlated with pain intensity.

Our research explored the changes of oscillatory activity in chronic pain patients with PHN and found enhanced gamma activity (40–70 Hz) (**Figure 1**). Although the narrow band low gamma power (30–48 Hz) exhibited a trend of increase in fibromyalgia patients (Lim et al., 2016) and a significant increase in rats with inflammatory chronic pain (Wang et al., 2016), our present study reports the significantly increased gamma power (40–70 Hz) of EEG in PHN patients for the first time. This finding is in line with previous studies which indicated enhanced gamma activities under ongoing and chronic pain condition (Peng et al., 2014; Schulz et al., 2015; Li et al., 2016; Lim et al., 2016). However, a recent research reported that the resting EEG in fibromyalgia patients did not show any significant changes in gamma power (30–48 Hz) (Fallon et al., 2018). This inconsistent result highlights the necessity of our study in a contrary position that the importance of expanding gamma frequency to a broad frequency band. Compared to chronic inflammatory pain model as in the previous report (Wang et al., 2016), chronic neuropathic pain was used in the current study. The common finding of enhanced gamma power indicates that the enhanced gamma activity is independent of the subtype of chronic pain.

Unlike previous studies in which enhanced theta or alpha in chronic pain patients were found (Sarnthein et al., 2006; Boord et al., 2008; Jensen et al., 2013; Van Den Broeke et al., 2013; González-Roldán et al., 2016; Fallon et al., 2018), our present study did not find any significant difference at theta and alpha frequency band in PHN chronic pain patients. This discrepancy may be explained by types and severity of neuropathic pain. For example, increased theta power was found in chronic pain patients with therapy-resistant syndromes (Sarnthein et al., 2006), and only chronic back pain patients with severe pain and root lesion showed a trend of increase of theta frequency (Schmidt et al., 2012). It suggests that severe pain is related with enhanced power at low frequency band. Obviously, pain in the PHN is neither severe nor resistant-therapy in our study. In addition, the peripheral neuropathic pain which we focused on in the present study is different from the central neuropathic pain as in other previous researches (Boord et al., 2008).

The gamma activity was strongly correlated with pain intensity. It is consistent with the findings in chronic pain study in rats (Wang et al., 2016). The correlation analysis demonstrated that severe pain had stronger gamma activity (**Figure 4**). In the current study, this relationship was further evidenced by one patient whose gamma power was reduced when the pain was relieved (**Figure 5**). Besides, the gamma power was also found to correlate with patient’s BDI and BAI scores. It is reasonable since the pain intensity was positively correlated with both BAI and BDI scores (**Figure 4**). As the concomitant symptoms of PHN, depression, and anxiety represent the affective aspects of chronic pain. This result is consistent with a previous finding that gamma power at narrow low frequency band in the DLPFC and orbitofrontal cortex was positively correlated with affective pain (Lim et al., 2016). The positive correlation between depression, anxiety, and gamma activities proved that the enhancement of gamma activity reflected the chronic pain. Interestingly, we also found that the difference of gamma power disappeared if the BAI variables were controlled, indicating that the enhanced gamma power was related to anxiety. Thus, these results suggest that the elevated gamma activity reflects the pain intensity and its symptoms of chronic pain, especially the pain-related anxiety symptom.

The sensor level and source level analysis found enhanced gamma activity in the dorsolateral and medial prefrontal areas (**Figures 2**, **3**), median and anterior cingulate and paracingulate cortices and cerebellum. The location information of gamma activity is comparable to that in the ongoing experimental pain (Schulz et al., 2015) and chronic pain (Lim et al., 2016; Wang et al., 2016) in which over activation of gamma band was discovered in the prefrontal areas. The prefrontal area has been implicated the non-sensory component of pain (Lorenz et al., 2003). Functional MRI showed that this area was more activated in chronic pain than acute pain (Apkarian et al., 2005). Elevated gamma activity in the prefrontal cortex and cingulate cortex in chronic pain indicates that non-sensory components such as cognitive and emotional aspects are more related to pathological chronic pain than the sensory aspect. Our results together with these reports highlight the importance of the cognitive aspect in chronic pain and support the theory of chronic pain in oscillatory levels.

In addition, we interestingly found for the first time that the gamma activity in the cerebellum was higher in PHN chronic pain patients. Cerebellum activation during pain was proposed to be related to integration of emotional and motor processing (Moulton et al., 2010). Gamma activity is believed to be a neural basis underlying integrating sensory information into a coherent perception (Jensen et al., 2007). Accordingly, altered gamma activity in cerebellum area in our study may suggest higher integration among pain-related factors including emotion and motion in the presence of chronic pain. Although the oscillation at gamma frequency band in cerebellum has been found *in vitro* and *in vivo*, the functional significance of these oscillations are still unclear (Dalal et al., 2013). Thus, the function of enhanced gamma activity in cerebellum under chronic pain condition needs further investigation.

We further explored the modulation of the phase of low frequency on the amplitude of gamma frequency (i.e., PAC) in PHN chronic pain patients. Though the theta phase seems have stronger modulation index, no statistical difference between chronic pain patients and healthy controls was found. It is different from transient acute pain in rats (Wang et al., 2011) and chronic inflammatory pain in rats which showed that theta phase entrained the gamma amplitude (Wang et al., 2016). The inconsistent findings of PAC in human and animals exhibit the species specificity, and highlights the importance of human study in the pain research field. In addition, theta-gamma PAC was significant between thalamus and cortical areas (LeBlanc et al., 2016), which could not be recorded by scalp EEG.

Enhanced gamma power at prefrontal and cingulate areas supports the TCD theory in chronic pain. According to TCD theory, the deafferentation inputs from the nerve lesion result in de-activation of low-threshold voltage-gated T-type Ca^2+^ channels in the thalamus. It then produces disinhibition of cortical GABAergic interneurons that exhibit enhanced gamma band activity in cortical areas received the projection from thalamus (Llinás et al., 2005). Besides, there is a positive correlation between gamma power and symptoms of pain. It is in line with the proposal of TCD that aberrant gamma activity is responsible for positive symptoms. Thus, our findings provide an evidence of abnormal gamma oscillation for TCD mechanism of chronic pain, and a potential manipulating oscillation for therapeutic intervention.

There were several potentially confounding factors need to be considered. For the consideration of treatment, the PHN patients were not free of medication. Though we excluded medicine that significantly influences the activity of high frequency oscillation, the confounding factors should be taken into consideration. According to previous researches, the medication including pregabalin, carbamazepine, or diazepam administration increased the oscillatory activity of the low frequency (delta, theta, and alpha band) (Salinsky et al., 2002; van Lier et al., 2004; Graversen et al., 2012). Because our study aims to explore the oscillatory activity at high frequency (gamma frequency band), we do not rule out cases with the medication that mainly affect the low frequency. Besides, high frequency activity seems decreased by the medication. It is opposite to our results, which shows increased gamma activity. Therefore, we believe the influence of medication is not significant or in an opposite way. Besides, there is relatively small sample size in our present study, which may reduce the statistic efficiency.

In summary, our results uncover the enhanced gamma oscillatory activity in prefrontal cortex and cerebellum in PHN patients which strongly correlates with pain intensity and pain relief. The altered gamma oscillatory activity in chronic pain provides evidence for TCD theory and a potential therapeutic target for oscillation intervention.

## Ethics Statement

This study was carried out in accordance with the recommendations of Measures for the Ethical Review of Biomedical Research Involving Humans, Ethics Committee of Peking University People’s Hospital. The protocol was approved by the Ethics Committee of Peking University People’s Hospital. All subjects gave written informed consent in accordance with the Declaration of Helsinki.

## Author Contributions

RZ, JW, HG, and YW designed the experiments. RZ, JW, and WQ performed the experiments. JW, F-YL, and MY analyzed the data. RZ, JW, and YW wrote the manuscript.

## Conflict of Interest Statement

The authors declare that the research was conducted in the absence of any commercial or financial relationships that could be construed as a potential conflict of interest.

## Footnotes

1 http://sccn.ucsd.edu/eeglab/
